# Study on the Moisture Absorption and Thermal Properties of Hygroscopic Exothermic Fibers and Related Interactions with Water Molecules

**DOI:** 10.3390/polym12010098

**Published:** 2020-01-04

**Authors:** Yi Cui, Shuyi Gao, Ruiyun Zhang, Longdi Cheng, Jianyong Yu

**Affiliations:** 1 Key Laboratory of Textile Science and Technology, Ministry of Education, College of Textiles, Donghua University, Shanghai 201620, China; 2150143@mail.dhu.edu.cn (Y.C.); 2160078@mail.dhu.edu.cn (S.G.); Ldch@dhu.edu.cn (L.C.); 2 Innovation Center for Textile Science and Technology, Donghua University, Shanghai 201620, China; yujy@dhu.edu.cn

**Keywords:** hygroscopic exothermic fiber, GAB model, moisture content, The net isosteric heats of sorption, Simultaneous thermal analysis

## Abstract

The aim of this paper is to study the hygroscopic behavior of hygroscopic exothermic fiber-based materials and to obtain a better understanding of the thermal performance of these fibers during the moisture absorption process. The temperature distribution of different kinds of hygroscopic exothermic fibers in the process of moisture absorption, observed by infrared camera, demonstrated two types of heating performance of these fibers, which might be related to its hygroscopic behavior. Based on the sorption isotherms, a Guggenheim-Anderson-de Boer (GAB) multi-layer adsorption model was selected as the optimal moisture absorption fitting model to describe the moisture absorption process of these fibers, which illustrated that water sorption capacity and the water–fiber/water–water interaction had a significant influence on its heating performance. The net isosteric heats of sorption decreased with an increase of moisture content, which further explained the main factor affecting the heat dissipation of fibers under different moisture contents. The state of adsorbed water and water vapor interaction on the fiber surface were studied by simultaneous thermal analysis (TGA-DSC) measurement. The percentage of bound and unbound water formation at low and high humidity had a profound effect on the thermal performance of fibers. It can therefore be concluded that the content of tightly bound water a strong water–fiber interaction was the main factor affecting the heating performance of fibers at low moisture content, and the content of loosely bound water reflected that water sorption capacity was the main factor affecting the heating performance of fibers at high moisture content. This was further proven by the heat of desorption.

## 1. Introduction

Advances in science and technology have seen the way we keep warm in the cold environment change from traditional heat insulation to active heat generation [1,2,3,4,5]. Hygroscopic exothermic fibers represent one of the active heat generation materials, and is becoming more and more popular today as it can meet the multiple requirements of winter clothes such as lightweight and warmth, comfort, functionality, and fashion. Further, based on relevant data, the amount of sweat discharges from skin is about 100 g/m^2^ per hour when the human body exercises, and 15 g/m^2^ per hour when the human body is at rest [6], which means there is a large amount of water evaporation through the skin at any time in order to provide an energy source for hygroscopic exothermic fibers that is meaningful to realize the heating function of textiles.

All materials generate heat when absorbing moisture, and absorb heat when releasing moisture. The heat of moisture absorption varies according to the materials, for example, the heat of moisture absorption in wool fiber with high moisture regain is larger than that in common synthetic fiber with low moisture regain, which indicates that the hygroscopicity of textile fibers has a significant influence on its heating performance. The common hygroscopic exothermic fiber materials are classified into two types: one is modified by using some monomers like acrylic acid, acrylate, acrylamide, and so on, while the other is modified using cellulose fiber as a matrix. One thing that these two types of fibers have in common is that they have a high moisture regain and release a lot of heat in the process of interacting with water molecules, which could be visually observed by infrared camera in the process of moisture absorption [7]. Hence, it seems that the water sorption capacity and water-fiber interaction are related to its heat dissipation. And the moisture adsorption behavior of high hygroscopic fibers and the causes of moisture absorption and heat generation of these fiber materials are worthy of discussion.

Nowadays, the basic theories that are usually used to explain the adsorption process include the monolayer adsorption theory based on the Langmuir equation, the multi-molecular layer adsorption theory based on the BET equation, the adsorption potential energy theory based on micropore filling, and the capillary condensation theory based on the Kelvin formula [8,9,10,11]. Moisture sorption isotherm reveals the equilibrium relationship between the moisture content of the material and water activity at a constant temperature and pressure, which can obtain the optimal model to predict the moisture adsorption process of fiber materials. Brunauer [12] classifies sorption isotherms according to their shape and processes, establishing five different types. Type 1 are Langmuir isotherms that present a characteristic increase in water activity related to the increasing moisture content. The first derivative of the plot increases with moisture content and the curves are convex upwards. Type 2 are sigmoidal sorption isotherms in which the curves are convex upwards at low water activity and inflection points usually occur near monolayer adsorption. It takes into account the existence of multilayers at the internal surface of a material. Type 3 sorption isotherms present convex downwards over the entire water activity range without curve inflection points. Type 4 sorption isotherms are similar to type 2 at low water activity and rise faster than type 2 at high water activity because of capillary condensation. Type 5 sorption isotherms are convex towards the water activity axis and there is an inflection point at high water activity.

In order to study the influence of fiber moisture absorption behavior on its heating performance, some mathematical models based on improved moisture absorption theory have been proposed to describe the moisture absorption isotherms for materials such as kinetic models based on monolayer sorption (BET model [13]), kinetic models based on multilayer sorption (GAB model [14]), semi-empirical models (the Ferro-Fontan model [15] and Henderson model [16]), and empirical models (Oswin model [17], Smith model [18], Peleg model [19]). In general, the GAB model is considered the most versatile due to its ability to cover a wider water activity range. The goodness of fit of any sorption model to the experimental equilibrium moisture content and relative humidity data should be judged based on statistical parameters like coefficient of determination (R2), squared error, and root mean squared error [20,21]. The analysis of sorption isotherm data by the application of thermodynamic principles provides the isosteric heat of sorption, which is a measure of energy that binds water molecules with the substrate surface, and it is also identified with the energy required to remove moisture from material.

In recent studies, water molecules restrained or closely contact with material surface have been paid considerable attention. Various kinds of water absorbed on hydrophilic polymers were analyzed using differential thermal analysis (DTA), differential scanning calorimetry (DSC), thermogravimetry (TG), and combined simultaneous thermal analysis (STA) [22,23]. There are three types of waters that exist in hygroscopic fiber, namely: freezing unbound water, freeze bound, and nonfreezing water [24]. Freezing unbound and freeze bound water do not create a strong interaction with the polymer matrix, which means there is no much energy released during the formation of weakly hydrogen bonds. Bound (nonfreezing) water refers to the water molecules that are directly hydrogen-bonded to the polymer chain, which releases significant energy during the formation of hydrogen bonds between the water molecular and hydrophilic groups in the polymer chain [25]. The heat generation performance of different hygroscopic fiber materials can be inferred from the state of water molecules in the fiber.

The main objective of this work is to reveal the cause of excellent heat dissipation of different hygroscopic exothermic fibers during the process of moisture absorption through the hygroscopic behavior of these fibers such as water sorption capacity of the fibers and the water–fiber/water–water interaction, which would lay a theoretical foundation for the development and application of such materials.

## 2. Materials and Experimental

### 2.1. Physical Properties Test and Preparation of Fiber Batting Samples

Vibration fiber fineness tester (XD-1, Shanghai, China) was used to determine the linear density of fibers in accordance to ISO 1973-1995. Each sample was measured 50 times and the average taken as the final result. The mechanical properties of the fibers, such as intensity and elongation, were tested in accordance to ISO 5079-1996 using tensile tester (XQ-1C, Shanghai, China). The gauge length and drawing speed were kept at 20 and 10 mm/min, respectively. Each sample was measured 50 times, and the average was taken as the final result. Further, the moisture regain of textile was obtained by using the drying method in accordance to GB/T 9995-1997. Each sample was measured 3 times and the average taken as the final result.

The tested basic physical properties of four kinds of hygroscopic exothermic fiber materials were listed in Table 1. The collagen protein viscose fiber, which was made by adding the collagen protein solution and the crosslinking agent to the spinning solution in a certain ratio, was provided by Taiyuan University of Technology. The modified polyacrylic acid (PAA) was a kind of acrylic fiber with hydrophobic finishing made by a special process composed of groups such as acrylic acid, sodium acrylate, and acrylamide, and it was prepared by Jie Yikang Chemical Technology Co., Ltd. (Shanghai, China). The modified polyacrylate (PEA) was purchased from TOYOBO STC Co., Ltd. (Osaka, Japan). Meanwhile, the modified polyacrylonitrile (PAN), which was grafted into a large number of hydrophilic groups on the macromolecular chain of polyacrylonitrile fiber, was provided by Rishu Technology Textile Co., Ltd. (Shanghai, China). All the fibers were opened into rolls by a carding machine to make uniform fiber battings (10 cm × 12 cm), then dried in a blower oven at 105 °C for 2 h and placed under atmospheric conditions (20 °C, 10% RH) for 24 h. 

### 2.2. IR Camera Test Analysis

IR camera (Tis75, Fluke, Everett, WA, USA) was used to determine the thermal properties of materials during the process of moisture adsorption. The fiber batting dried in a desiccator with silicone was humidified by a humidifier under standard atmospheric conditions (20 °C, 65%RH). Record the temperature changes of fiber batting in an hour using IR camera.

### 2.3. Scanning Electron Microscopy (SEM) and FT-IR Analysis

SEM micrographs of fiber surface topography of fibers were taken using a scanning electron microscope (Quanta250, Hillsboro, OR, USA). It was operating at 10 kV, 20 °C, and RH 65%. Prior to SEM evaluation, the samples were coated with a thin layer of gold by means of a plasma sputtering apparatus.

FT-IR spectroscopic analysis was used to determine the chemical functional groups of fiber samples. The samples dried at 105 °C for 2 h and placed for two days in a desiccator with silicone (20 °C, 10%RH) were investigated using a Nexus 670 Spectrometer (Thermo Nicolet, Waltham, MA, USA) using Attenuated Total Reflectance (ATR) using ZnSe crystal. The spectra obtained were the results of 10 scans over a range of from 600 to 4000 cm^−1^ with a resolution of 4 cm^−1^, followed by baseline correction, and smoothing prior to further analysis.

### 2.4. Determination of Sorption Isotherms and Model Fitting

The sorption isotherms for the different kinds of hygroscopic exothermic fibers were determined using the static gravimetric method. A total of 6 saturated salt solutions were used to maintain constant relative humidity (11%, 35%, 49%, 79%, 89% and 98%, respectively) at 4 temperatures, namely 20, 25, 30, and 35 °C. The salts used were LiCl, MgCl_2_·6H_2_O, K_2_CO_3_, NaCl, KCl, and K_2_SO_4_ [26]. Since the water activity was approximated as the ratio of the partial pressure of water vapor in the solution to the partial pressure of pure water vapor at a certain temperature, the relative humidity (RH) could be replaced by the water activity (aw) as follows [27]:

aw = RH/100(1)

Weigh the fiber sample dried in a blower oven at 105 °C for 2 h and record the weight of the initial fiber as *w*1. Then, place it in a desiccator with 100 mL of different supersaturated salt solution at the bottom, and place the desiccator at a temperature controlled at 20, 25, 30, and 35 °C (±1 °C). In the constant humidity chamber, the weight of the sample was accurately measured on a regular basis until the difference between the two measurements was not more than ±0.001 g, and all fiber samples were measured three times. Record the weight of the moisture-absorbed fiber as *w*2. The equilibrium moisture content m was calculated using Equation (2):

(2) $$m=\frac{w2-w1}{w1}*100\%$$

where *w*1 was the weight of initial fiber and *w*2 was the weight of fiber at moisture sorption equilibrium.

The sorption isotherm models used to fit experimental data were shown as follows. The parameters of models used were estimated with cftool nonlinear function fitting of Matlab software (R2015b, MathWorks, Natick, MA, USA) in order to select the best correlation and to improve the analysis of the experimental data.

Guggenheim-Anderson-de Boer (GAB) model [14]:

The GAB model was a refinement of Langmuir and BET theories of physical adsorption, and it was expressed as it was expressed in Equation (3):

(3) $$m=\frac{m0*C*K*\mathrm{aw}}{\left(1-K*\mathrm{aw}\right)\left(1-K*\mathrm{aw}+K*C*\mathrm{aw}\right)}$$

where *m*0 denotes the monolayer moisture content, while C and K are the adsorption constants, which were related to the energies of interaction between the first and the further absorbed molecules at the individual sorption sites.

Oswin model [17]:

This is an empirical model that constitutes a series expansion for sigmoid shaped curves and is was described by Equation (4):

(4) $$m=A*{\left(\frac{\mathrm{aw}}{1-\mathrm{aw}}\right)}^{B}$$

where A and B are constants.

Smith model [18]:

This is an empirical model used to postulate that there were two fractions of water that were absorbed onto a dry surface: the first fraction exhibited a higher condensation heat than the normal and it would be expected to follow the Langmuir model. Smith based his model on the second fraction, which only could be formed after the first fraction had been absorbed. Smith considered that the second fraction consists of multiple layers of condensed water molecules, and the model could be written as Equation (5):

(5) m=P+Qln(1−aw)

where P represents the quantity of water in the first absorbed fraction and Q is the quantity of water in the multilayer moisture fraction.

Peleg model [19]:

This is an empirical sigmoidal isotherm model that was developed to circumvent the assumption of a specific water content for monolayer coverage, as is the case in the BET and GAB models. This double power law model assumes that sorption comprises two mechanisms: (i) the lower the aw, the lower the sorption rate, and (ii) at higher aw values, there is an increase in free condensation. This model has four parameters and can be described in Equation (6):

(6) $$m=m1{*\mathrm{aw}}^{n1}+m2{*\mathrm{aw}}^{n2}$$

where *m*1, *m*2, *n*1, and *n*2 are constants, *n*1 < 1 and *n*2 > 1. The model did not have a monolayer incorporated into it.

The goodness of the fit of each sorption model to the experimental equilibrium moisture content and water activity data was evaluated based on statistical parameters like coefficient of determination (R2), the sum of squared error (SSE), and root mean squared error (RMSE):

(7) $$\mathrm{SSE}=\overset{n}{\underset{i=1}{\sum }}{\left(m_{\exp}-m_{\mathrm{cal}}\right)}^{2}$$

(8) $$\mathrm{RMSE}={\left[\frac{1}{n}*\overset{n}{\underset{i=1}{\sum }}{\left(m_{\exp}-m_{\mathrm{cal}}\right)}^{2}\right]}^{1/2}$$

where *m*_exp_ is the experimental water content, *m*_cal_ is the calculated water content, and n is the number of data points.

The net isosteric heat of sorption (*q*_st_) for specific equilibrium moisture content (*m*) referred to the energy released during the process of moisture adsorption in the material surface, which reflected the force between the water molecules and the solid matrix at the adsorption site. This could be calculated from the experimental data using the Clausius–Clapeyron Equation [28]:

(9) $$q_{\mathrm{st}}=-R{\left[\frac{\partial \left(\ln\left(\mathrm{aw}\right)\right)}{\partial \left(\frac{1}{T}\right)}\right]}_{m}$$

where *R* was gas constant (8.314 J mol^−1^ K^−1^) and *T* was temperature. The net isosteric heat of water vapor adsorption (*q*_st_) was determined by plotting the sorption isotherm as ln(aw) vs. 1/*T* for fixed values of moisture content based on Equation (4) and then determined the slope of the resulting straight line which was equal to –*q*_st_/*R* [29].

### 2.5. TGA-DSC Analysis

Different hygroscopic exothermic fibers-based materials were analyzed by combined thermo gravimetric and differential scanning calorimeter instrument (TGA–DSC, STA409PC, Netzsch, Serb, Bavaria, Germany) to determine the heat of desorption and vaporization (J/g) and the amount and the form of water availability at different relative humidity. Samples were equilibrated at desiccators with different relative humidity (RH = 11%, 35%, 49%, 79%, 89%, 98%) until the constant mass of the sample was established and recorded. Then, the samples were heated at a heating rate of 10 °C/min and nitrogen was used as the purge gas at a flow rate of 10 mL/min. The weight loss and heat flow of the samples were recorded for the temperature range 40–250 °C.

## 3. Results and Discussion

### 3.1. Thermal Management Test

**Figure 1:**
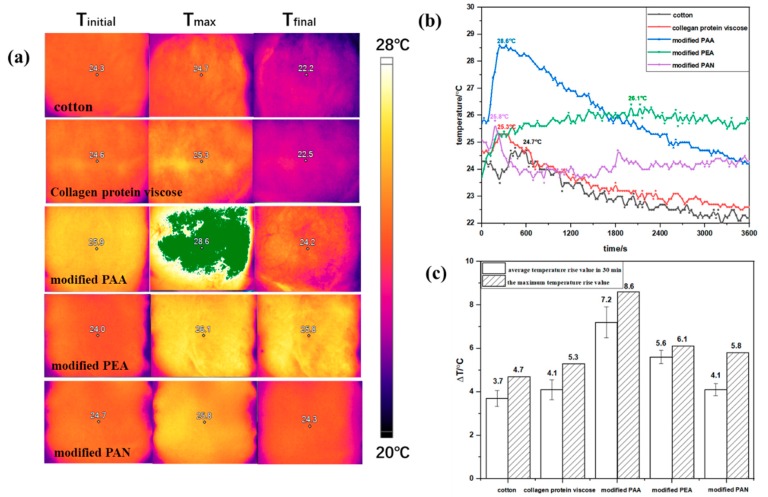
Thermal properties of different fiber battings during moisture absorption. (**a**) IR images showing the temperature distributions of different fiber battings at initial temperature point, maximum temperature point and final temperature point during the moisture absorption. (**b**) Temperature curve of fiber battings during moisture absorption. (**c**) Average temperature rising value in 30 min and the maximum temperature rising value during moisture absorption. Note: The green part of Figure 1a indicates that the temperature of modified PAA batting exceeds the limit of set temperature of 28 °C.

IR camera test systems were built to investigate the thermal properties of collagen protein viscose, modified PAA, modified PEA, and modified PAN. In order to compare the difference in moisture absorption and exothermic property between four fibers and ordinary fibers, the IR camera test of cotton fiber was used as a comparison. The measurement details (temperature distribution on fiber battings) are shown in Figure 1 and Figure S1. In general, the temperature rise values of four hygroscopic exothermic battings were higher than cotton bating, which illustrated the better heating performance of these kind of materials than ordinary fibers. Further, for the cotton batting, collagen protein viscose batting, and modified PAA batting, the temperature rose with the moisture absorption until reaching the maximum, after which they exhibited a decreasing trend. The fiber materials with a high moisture regain rate, like modified PEA and modified PAN, released heat evenly and constantly as the moisture absorption process continued compared to other fiber battings. As can be observed in Figure 1c, the average temperature rising value in 30 min and the maximum temperature rising value [30] of modified PAA batting were the highest in these fiber batting materials, which were 7.2 and 8.6 °C, respectively. It seemed that there were two types of heating performance of fiber battings during the moisture adsorption process, which might be related to the different moisture adsorption behaviors of these fibers.

### 3.2. Surface Characterization

**Figure 2:**
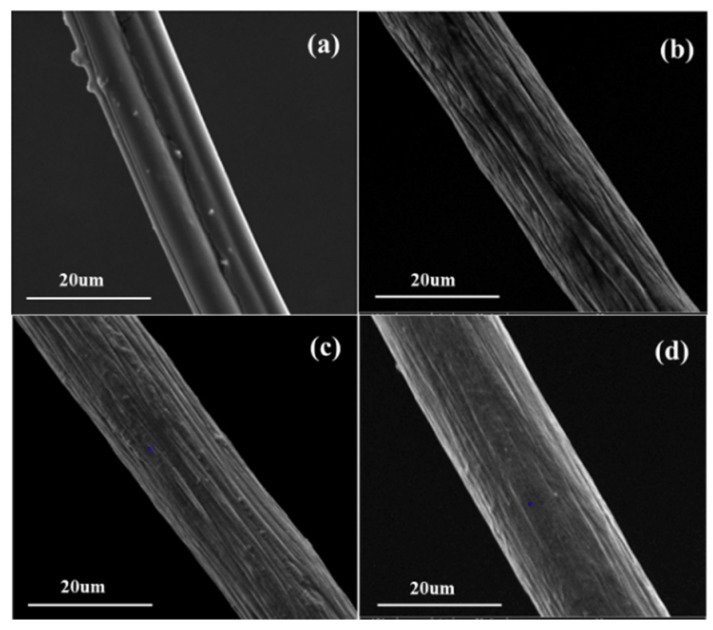
SEM surface image of (**a**) collagen protein viscose, (**b**) modified PAA, (**c**) modified PEA, (**d**) modified PAN.

Scanning electron microscope (SEM) images of different samples are illustrated in Figure 2. (surface and cross section image of all fiber samples were provided in the Supplementary Figure S2 supplementary material). The surface of the collagen protein viscose fiber was relatively smooth but some granular objects adhered, which might be the residual protein granules after the modification. Figure 2b,c illustrates the obvious crimple at the surface and the bark-like grooves in the axial direction distributed deeply and unevenly. This modification benefited the surface roughness and specific surface area of the fiber, which facilitated the moisture adsorption and transfer. There were shallow and relatively fine grooves on the surface of modified PAN, indicating the small specific surface area of this fiber.

**Figure 3:**
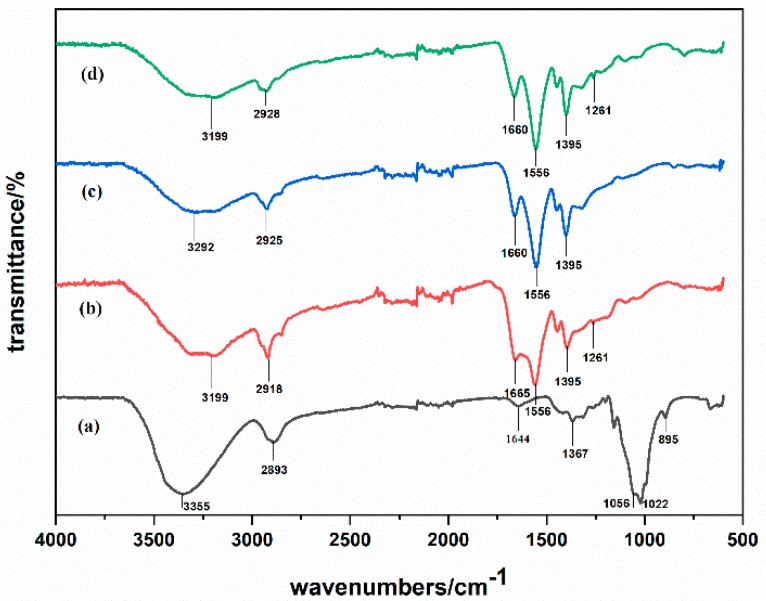
The FT-IR of different hygroscopic exothermic fiber samples: (**a**) collagen protein viscose (**b**) modified PAA (**c**) modified PEA (**d**) modified PAN.

It could be seen that the absorption peak positions of modified PAA, modified PEA, and modified PAN were basically the same from Figure 3. There was a broad peak at 3200–3300 cm^−1^, indicating that these three fibers might contain association –OH or association –NH. The peaks at 1660 and 1665 cm^−1^ were the stretching vibration of C=O in the amide group. The absorption peak intensity was consistent at 1556 cm^−1^ (corresponding to the antisymmetric stretching vibration of –COO–) and 1395 cm^−1^ (corresponding to the symmetric stretching vibration of –COO–), which indicated that all these three fibers contained carboxylate group. The medium intensity adsorption peak of secondary amide group at 1261 cm^−1^ indicated the presence of –CONH group. For collagen protein viscose fiber, the –OH, –NH stretching vibration absorption peak at 3355 cm^−1^ showed that the fiber contained –OH, –NH_2_ group. Furthermore, the spectra of this fiber exhibited amide I characteristic absorption peak at 1644 cm^−1^, –OH bending plane vibration around 1367 and 1056 cm^−1^, and S–O vibration absorption peak was around 1022 cm^−1^.

It was known that the structure and mechanical/physical properties of polymer materials would change after absorbed water, which was related to the degree of chemical or physical association between water and polymer materials. Each polar group in different polymer materials could be connected to a different amount of non-crystalline bonded water. One hydroxyl group could be bonded to one non-crystalline bonded water, one amide group could be connected to 4.2 non-crystalline bonded water and each C=O group could be connected to two amorphous bonding water [31]. Thence, the moisture sorption capacity of modified PAA, PEA, and PAN, which mainly contained carboxyl groups, carboxylate groups, amide groups, and so on was higher than that of collagen protein viscose, which mainly contained hydroxyl groups. 

### 3.3. Moisture Adsorption Equilibria and Isosteric Heats

In order to study the influence of fiber moisture absorption behavior on its heating performance, four moisture absorption models were selected to fit its moisture absorption process. The evaluation of the four sorption models were obtained and shown in Table 2. It could be seen that both GAB and Peleg models had a higher determination coefficient (R^2^›0.99), smaller sum of squared error (SSE‹0.003), and smaller root mean squared error (RMSE‹0.03) at four different temperatures (20, 25, 30, and 35 °C). Though peleg model had good predictability, the model was only an empirical pure mathematical simulation which couldn’t explain the hygroscopic properties of the fibers. At the same time, the SSE and RMSE of the Peleg model were larger than the GAB model and the GAB model could reflect the monolayer moisture content and adsorption energy constant during moisture adsorption. Accordingly, the GAB model was selected as the optimal moisture absorption model for these hygroscopic exothermic fibers.

**Figure 4:**
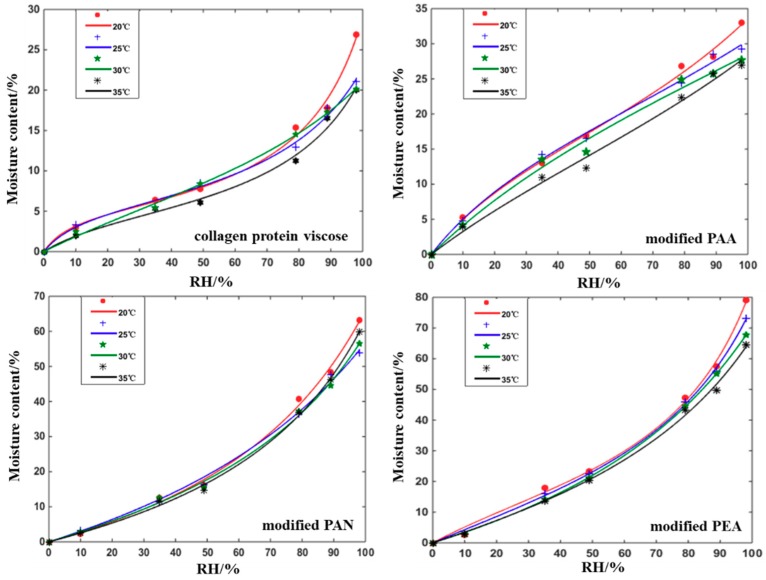
Experimental data and Guggenheim-Anderson-de Boer (GAB)-predicted GAB-predicted water vapor adsorption isotherms at different temperature for different fiber samples (symbols are experimental data and solid lines are the model predictions).

Moisture adsorption isotherms at different temperatures (20, 25, 30, and 35 °C) for different fiber samples were shown in Figure 4. In general, the equilibrium moisture content of the four fibers exhibited a decreasing trend with the increase of temperature. This was because the higher temperature would increase the activation energy of the water molecules and reduced the binding force with the surface of materials, which caused the water molecules to escape from the binding site and decreased the equilibrium water content [32]. The GAB model demonstrated an excellent fit to the experimental adsorption data, as shown in the Figure 4. The highest adsorption was observed for the modified PEA with moisture content of 79 wt. % at RH = 98%. The isotherms of collagen protein viscose and modified PAA formally belong to Type 2 and the isotherms of modified PEA and modified PAN belong to Type 3 [12], which corresponded to the two types of heating performance of fiber battings during the moisture adsorption process shown in Figure 1. In this study, collagen protein viscose and modified PAA showed lower water vapor adsorption capacities in comparison to modified PEA and modified PAN. It seemed that modified PEA and modified PAN demonstrated good capacity to store the water vaper, which could facilitate the moisture adsorption and heat release.

**Figure 5:**
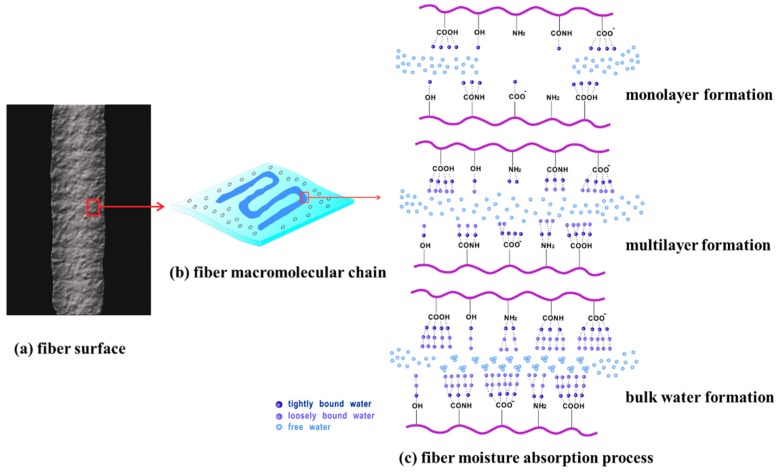
Schematic diagram of moisture absorption process of hygroscopic exothermic fibers.

It was concluded that the GAB model was a suitable absorbent model to explain the moisture adsorption process of these hygroscopic exothermic fiber materials. The GAB multi-layer adsorption model was improved, based on the BET multi-layer adsorption model [33], which had one more parameter K related to the adsorption heat of the multi-molecular layer, and had a wider application environment than the BET model. The moisture absorption processes of four kinds of hygroscopic exothermic fibers conformed to the multi-molecular layer adsorption rule, as shown in Figure 5. At low moisture content, there were a large number of hydrophilic groups as active adsorption sites on the fiber surface and these had directly formed strong intermolecular force with water vapor in the moist air, and the moisture content was the monolayer water content of the fiber material. At high moisture content, as the water molecular coverage on the surface of the fiber material increased, water molecules that formed a strong interaction with the hydrophilic group formed an intermolecular force with other water molecules in the moist air until the adsorption equilibrium of the second layer of water molecules was reached. Then, the third layer, the multilayer moisture adsorption layer was formed. In addition, there was some bulk water directly attached to the voids and holes in the surface of the fiber material.

Monolayer moisture content (*m*0) and constants K and C were derived from the GAB model for different hygroscopic exothermic fibers, as summarized in Table 3. The monolayer moisture content m_0_ was the minimum moisture content covering hydrophilic sites on the material surface, the water content of the monolayer increased with the rise of temperature, which was related to the hydrophilicity of the fiber. Within the 20~30 °C temperature range, the relative motion of the macromolecular chains of the fibers increased as the temperature rose slowly, which caused more adsorption sites containing hydrophilic groups to be exposed on the surface of the molecular chain and to combine with more water molecules in the moist air to form stronger intermolecular force [34,35,36]. However, the kinetic energy of the water molecules with a weak binding force to the surface groups was increased when the temperature continued to rise, which led the water molecules to detach from the binding site of the fiber surface and reduced the water content of the monolayer. C is a measure of the strength of water molecule binding to primary binding sites. The larger C, the stronger water–fiber interaction in the monolayer, and the larger the difference in enthalpy between the monolayer molecules and multilayer molecules. It can be seen from the Table 3 that the binding force with water molecule of modified PAA and collagen protein viscose was relatively larger than that of modified PEA and modified PAN. So, the thermal performance of modified PAA and collagen protein viscose was better at the beginning of moisture absorption without a stable monolayer. K was related to the heat of multilayer sorption and was usually between the energy values of the molecules in the monolayer and liquid water energy of vaporization. When K approached one, there was almost no distinction between multilayer molecules and liquid molecules [37]. From the GAB analysis, it seemed that modified PAA with a large moisture regain had a lower K value compared to the other fibers, which could thus infer that there was a small amount of bulk water in the fibers.

**Figure 6:**
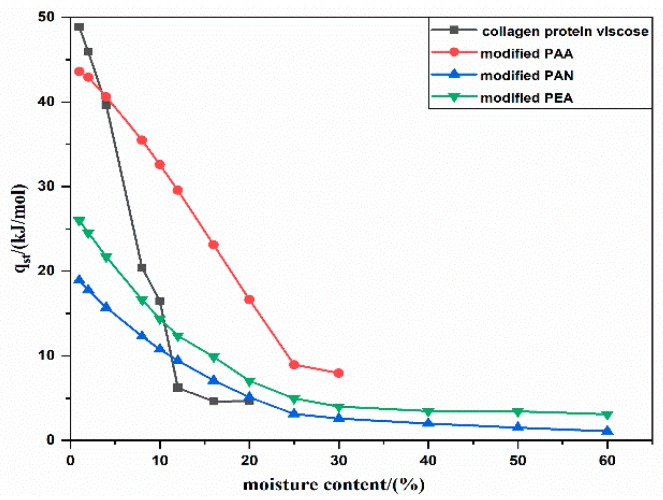
Effect of moisture content on the net isosteric heat of adsorption for different hygroscopic exothermic fibers.

The net isosteric heats of sorption (*q*_st_) exhibited a decreasing trend with the increase of moisture content, as shown in Figure 6. At low moisture content, a large amount of water molecules was tightly bound to the fiber materials, corresponding to high energy of interactions and high net isosteric heats. The values of *q*_st_ decreased by continuing adsorption because of the reduced interactions between monolayer water vapor and second and subsequent layers of water vapor formed on the monolayer. The net isosteric heats for collagen protein viscose (*q*_st_ = 48.84 kJ/mol), modified PAA (*q*_st_ = 43.6 kJ/mol), modified PEA (*q*_st_ = 26.01 kJ/mol) and modified PAN (*q*_st_ = 18.92kJ/mol) were obtained at the moisture content of 1.0%. Modified PEA and modified PAN fibers showed relatively lower values of C (i.e., low strength of water molecules bound to the surface), which was in a better agreement with the lower values of net isosteric heat at the beginning of moisture absorption. Further to that, modified PAA and collagen protein viscose fibers showed relatively high C value corresponding to the higher net isosteric heat, as expected. At high moisture content, *q*_st_ of modified PEA and modified PAN demonstrated that these two kinds of fibers still had the adsorption heat, which explained the continuously and even heat dissipation during moisture absorption shown in Figure 1. This indicated that the number of hydrophilic groups and the strength of binding force between water molecules and hydrophilic groups were the main factors affecting the heat release of materials with lower moisture contents, like modified PAA and collagen protein viscose fibers. Further, the moisture absorption capacity was the main factor affecting the heat release of materials with high moisture content, like modified PEA and modified PAN fibers, since there was no additional net heat generation in those fibers without high moisture absorption capacity.

### 3.4. Thermal Properties Analysis

**Figure 7:**
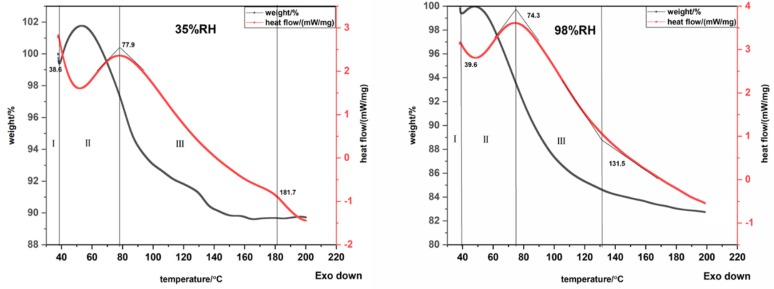
TGA-DSC analysis of absorbed water state on modified PAA at 20 °C, 35%RH and 20 °C, 98%RH.

The state of water molecules of hygroscopic exothermic fibers in accordance with the multi-molecular layer adsorption rule was divided into three types: tightly bound water (water molecules directly combined with groups of fiber surface), loosely bound water (second and subsequent layers of water vapor formed on the monolayer), and free water (bulk water directly attached to the surface of the fiber materials). In this study, different forms of adsorbed water present on high hydrophilic fiber materials were analyzed using TGA-DSC. Three different regions were identified on the TGA-DSC curves (Figure 7). These regions were defined by inflection points from the rate of mass loss and heat flow curves, and this helped to establish insights into the form of adsorbed water [22,23,38] (the identified regions from TGA-DSC curves of different hygroscopic exothermic fibers are shown in Supplementary Figure S3). The region I was assigned to the evaporation of free water from the crucible and water on the surface of the fiber. The region II could be assigned to loosely bound water. The region III was considered to be related to evaporation of tightly bound water.

**Figure 8:**
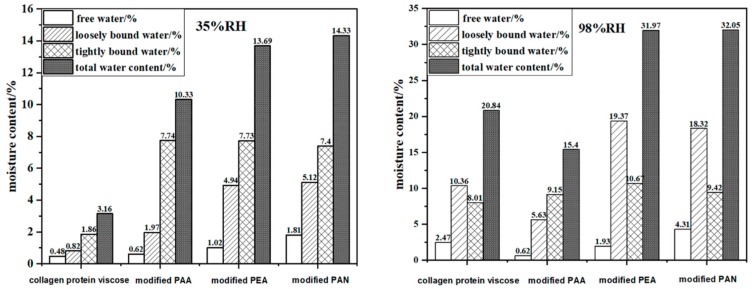
Moisture contents of different water formations on samples at 20 °C for 35%RH and 98%RH determined by TGA–DSC analysis.

The weight of modified PAA fiber exhibited a decreasing trend with the increase of temperature at the beginning, as shown in Figure 7. Then, the weight of fiber increased which was attribute to baseline drift due to small sample weight. The moisture content of different water formations of samples at 98%RH were higher than that at 35%RH, except for the free water content of the modified PAA, as shown in Figure 8. And the proportion of different types of water on samples at 20 °C for 35%RH and 98%RH was shown in Supplementary Table S1. At low relative humidity, the ratio of the tightly bound water and the total water content of the four kinds of fibers was the highest, indicating that the moisture inside the fibers was mainly in the form of tightly bound water. The main factors affecting the heating performance included the content of tightly bound water and strong intermolecular attraction force between the fiber’s hydrophilic group sites and water vapor molecules, which illustrated that the higher intermolecular attraction force, the larger thermal energy was released during moisture absorption. At high relative humidity, the ratio of loosely bound water and total water content was the highest, except for modified PAA, indicating that the moisture inside the fibers mainly existed in the form of loosely bound water. Compared to collagen protein viscose fibers, the loosely bound water in the modified PEA and modified PAN accounted for more than 50% of the total bound water, indicating that, due to its good water storage capacity, more water molecules were adsorbed on the fiber surface and more heat energy was released evenly and continuously. At the same time, too much free water content in the fiber would cause a large amount of heat to be absorbed during the moisture desorption process and reduce the overall exothermic effect of the fiber. The free water content in collagen protein viscose fibers and modified PAN accounted for more than 10% of the total fiber water content. Therefore, its final exothermic effect was worse than that of modified PEA.

The desorption enthalpy values were determined from the endothermic peaks obtained from the TGA–DSC measurements for four different kinds of fibers, as shown in Table 4. The heat required to remove the water from the fiber surface increased as the moisture content increased, and at low relative humidity, the content of tightly bound water and the bonding strength formed inside the fiber had an important effect on moisture desorption. The greater the content of tightly bound water and the stronger the binding force, the more energy was required for moisture desorption. Hence, the desorption heat of modified PAA was the largest compared to other hygroscopic exothermic fibers. At high relative humidity, the water molecules were loosely associated with the fiber surface by forming multilayers. The energy required to remove water molecules attached to fiber surface was higher than the energy which held water molecules in the liquid phase [39]. At this time, the content of loosely bound water determined the desorption heat. The lower content of loosely bound water of modified PAA and collagen protein viscose indicated a lower desorption heat than the other two kinds of fibers.

In general, hygroscopic exothermic process of hydrophilic fiber was roughly divided into two stages. In the first stage, at the lower moisture content, the state of absorbed water was mainly presented in the form of tightly bound water combined with hydrophilic groups of macromolecular chains and the heat was mainly derived from the content and the intermolecular attraction force of tightly bound water. The more tightly bound water content indicated the larger thermal energy released. Meanwhile, the stronger intermolecular attraction force evinces the better heat release effect during moisture absorption. At high moisture content, the hydrophilic group adsorption sites were almost occupied by water molecules as the moisture content inside the fiber materials increased, and the main state of water was the loosely bound water combined with tightly bound water. Despite the weak water–water interaction and small thermal energy production, plenty of water–water interaction occurred in the multi-molecular layer, accumulating a large amount of energy due to the moisture absorption capacity of the fiber materials. Hence, the heat was primarily related to the content of loosely bound water. At the same time, the free water in fiber materials should be noted due to its evaporation and absorbed heat. Therefore, the most effective way to improve the heating performance of this kind of fiber was to graft a large number of hydrophilic groups with strong binding force to water molecules and reduce the fiber crystallinity to improve its loosely bound water content. Furthermore, a waterproof finishing agent was applied to the surface in order to prevent the formation of a large amount of bulk water.

## 4. Conclusions

In summary, the temperature distribution of different kinds of hygroscopic exothermic fibers in the process of moisture absorption observed by infrared camera illustrated the better heating performance of these kind of materials than ordinary fibers and demonstrated the two types of heating performance of these fibers which might be related to its hygroscopic behavior. Moisture absorption equilibria and isosteric heats were obtained to study the influence of fiber moisture absorption behavior on its heating performance. Based on the experimental sorption isotherms measured at 20, 25, 30, and 35 °C, the GAB multi-layer adsorption model was selected as the optimal fitting model to describe the moisture adsorption process of these fibers, which illustrated the water sorption capacity and that water–fiber/water–water interaction had a significant influence on its heating performance. The net isosteric heats of adsorption, reflected in the degree of absorption between the water molecules and the solid matrix at the adsorption site for the fiber samples, decreased with an increase in the moisture content. Therefore, the strength of water–fiber surface interaction and energy release was gradually decreased with the increase of multilayers. Water formation and desorption energies were determined for different samples using TGA–DSC analysis. The heat of desorption was higher with the increase of moisture content in fiber materials and the percentage of bound and unbound water formation at low and high humidity had a profound effect on the thermal performance of fibers, which revealed the cause of heat release of these fibers during the process of moisture absorption. Therefore, the most effective way to improve the heating performance of this kind of fiber was to graft a large number of hydrophilic groups with strong binding force to water molecules, and to reduce the fiber crystallinity to improve its loosely bound water content. Furthermore, a waterproof finishing agent was applied to the surface to prevent the formation of a large amount of bulk water.

## Figures and Tables

**Table 1:** Physical properties of hygroscopic exothermic fiber samples.

Fiber Samples	Linear Density/(D)	Length/(mm)	Intensity/(cN/dtex)	Elongation/(%)	Moisture Regain/(%)	Grammage/(g/m^2^)	Thickness/(mm)
Collagen protein viscose	1.5	38	2.34	19.77	12.05	2.23	8
Modified PAA	3.1	51	0.67	12.85	29.64	2.23	11
Modified PEA	1.3	33	0.72	22.4	35.36	2.63	9
Modified PAN	2.5	51	0.58	39.94	26.71	2.65	8

**Table 2:** Evaluation of different models for four kinds of hygroscopic exothermic fibers.

Model	T/°C	Collagen Protein Viscose			Modified PAN			Modified PEA			Modified PAA		
		R^2^	SSE	RMSE	R^2^	SSE	RMSE	R^2^	SSE	RMSE	R^2^	SSE	RMSE
**GAB**	**20**	0.9951	0.000261	0.008071	0.9972	0.001001	0.01582	0.997	0.001562	0.01976	0.997	0.000273	0.008261
	**25**	0.993	0.000249	0.007899	0.9997	8.19 × 10^−5^	0.004526	0.9987	0.000594	0.01219	0.9963	0.00029	0.008512
	**30**	0.9978	7.96 × 10^−5^	0.00446	0.9977	0.000668	0.01293	0.9998	7.56 × 10^−5^	0.004349	0.9924	0.000544	0.01167
	**35**	0.993	0.000234	0.007643	0.9985	0.000469	0.01083	0.998	0.000754	0.01373	0.9923	0.000523	0.01143
**Oswin**	**20**	0.9764	0.001254	0.01584	0.9151	0.03061	0.07825	0.942	0.03044	0.07802	0.9152	0.007765	0.03941
	**25**	0.9423	0.002065	0.02032	0.9209	0.02568	0.07166	0.9276	0.03387	0.08231	0.8883	0.008761	0.04186
	**30**	0.9146	0.003034	0.02463	0.9143	0.0247	0.07028	0.9086	0.03856	0.08782	0.874	0.008996	0.04242
	**35**	0.9377	0.002081	0.0204	0.9198	0.02572	0.07172	0.9163	0.03102	0.07877	0.8821	0.008046	0.04011
**Smith**	**20**	0.968	0.001702	0.01845	0.9358	0.02315	0.06804	0.9499	0.02626	0.07248	0.8085	0.01753	0.05922
	**25**	0.9094	0.00324	0.02546	0.9413	0.01904	0.06171	0.9363	0.02981	0.07722	0.7472	0.01983	0.06298
	**30**	0.878	0.004337	0.02945	0.9267	0.02112	0.06499	0.9191	0.03409	0.08257	0.7406	0.01852	0.06086
	**35**	0.9355	0.002154	0.9355	0.943	0.01828	0.06047	0.9229	0.02859	0.07561	0.7964	0.01389	0.05271
**peleg**	**20**	0.994	0.000318	0.0103	0.9927	0.002648	0.02971	0.9987	0.000669	0.01494	0.9955	0.000408	0.01166
	**25**	0.9428	0.002046	0.02612	0.9926	0.002408	0.02833	0.9968	0.001367	0.02135	0.9955	0.000355	0.01088
	**30**	0.9993	2.45e-05	0.002857	0.9915	0.002452	0.02859	0.9963	0.001552	0.02275	0.9932	0.000485	0.01272
	**35**	0.9466	0.001783	0.02438	0.9987	0.000411	0.0117	0.9938	0.002307	0.02773	0.9939	0.000414	0.01175

**Table 3:** Parameters of four kinds of hygroscopic exothermic fibers after fitted by the GAB model.

Fiber Materials	*T*/°C	*m*0/%	C	K
collagen protein viscose	20	5.20	14.05	0.8238
	25	5.88	11.14	0.7488
	30	10.41	2.98	0.5781
	35	5.05	6.36	0.7785
modified PAN	20	39.8	1.03	0.6215
	25	27.6	1.41	0.6773
	30	32.55	1.33	0.6225
	35	29.92	1.20	0.6662
modified PEA	20	22.14	3.33	0.7587
	25	27.09	2.29	0.7028
	30	51.19	1.12	0.5686
	35	35.25	1.56	0.6208
modified PAA	20	19.38	6.08	0.5032
	25	23.5	6.49	0.3822
	30	35.73	5.39	0.2281
	35	23.43	3.53	0.4113

**Table 4:** Heats of desorption from TGA-DSC analysis.

Relative Humidity/%	Heats of Desorption of Fiber Samples (J/g)			
	Collagen Protein Viscose	Modified PAA	Modified PEA	Modified PAN
35	16.7	140.7	95.0	85.3
98	191.5	243.6	501.1	477.8

## Supplementary Materials

The following are available online at https://www.mdpi.com/2073-4360/12/1/98/s1, Figure S1: Temperature distribution of different fiber batting during the moisture adsorption; Figure S2: surface images and Cross section images of (a)collagen protein viscose, (b)modified PAA, (c)modified PEA, (d)modified PAN; Figure S3: TGA-DSC analysis of absorbed water state on different fibers at 20 °C, 35% RH and 20 °C,98% RH; Table S1: Table S1 Moisture contents of different water formations on samples at 20°C for 35% RH and 98% RH determined by TGA–DSC analysis.
