# Supplementary material for: Study on the Moisture Absorption and Thermal Properties of Hygroscopic Exothermic Fibers and Related Interactions with Water Molecules
Source: Polymers (Basel). 2020 Jan 4;12(1):98. doi: 10.3390/polym12010098 (PMC7023626; doi:10.3390/polym12010098)
Supplement: Supplementary file 1 [file polymers-12-00098-s001.pdf]

Supplementary Information

Study on the moisture adsorption and Thermal Properties of Hygroscopic Exothermic Fibers and Related Interactions with Water Molecules

1. IR camera test

The temperature distribution of different fiber batting materials in an hour was measured during the process of humidification was shown in Fig. S1.

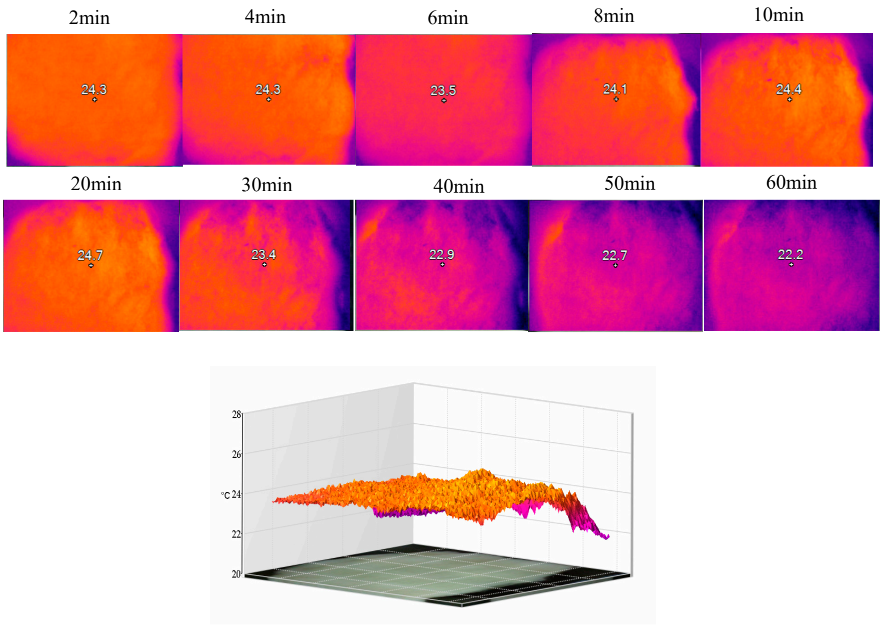

a. Temperature distribution of cotton batting during moisture adsorption

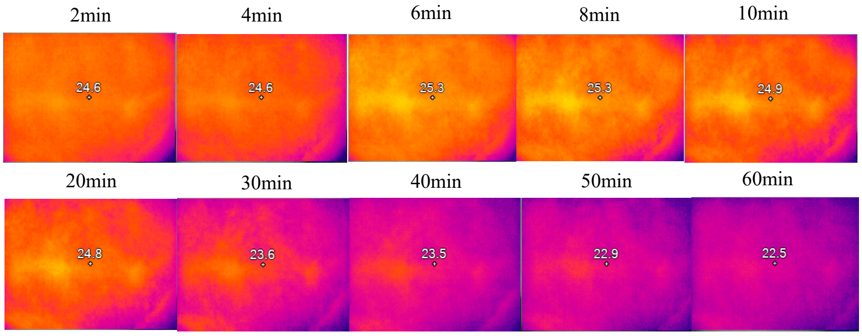

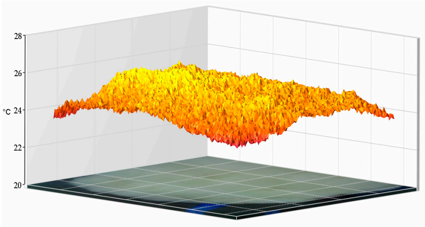

b. Temperature distribution of collagen protein viscose batting during moisture adsorption

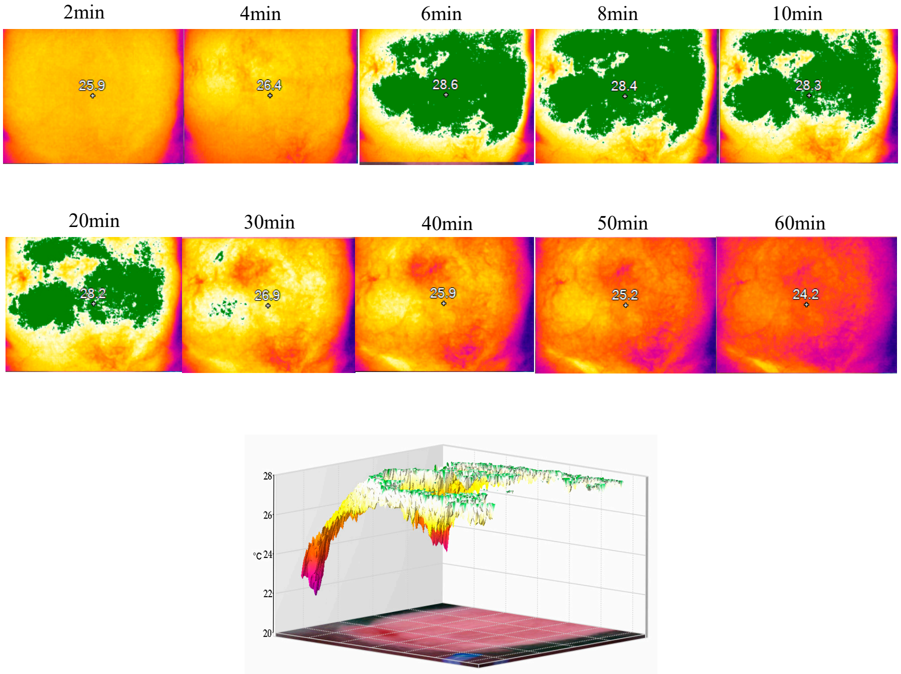

c. Temperature distribution of modified PAA batting during moisture adsorption

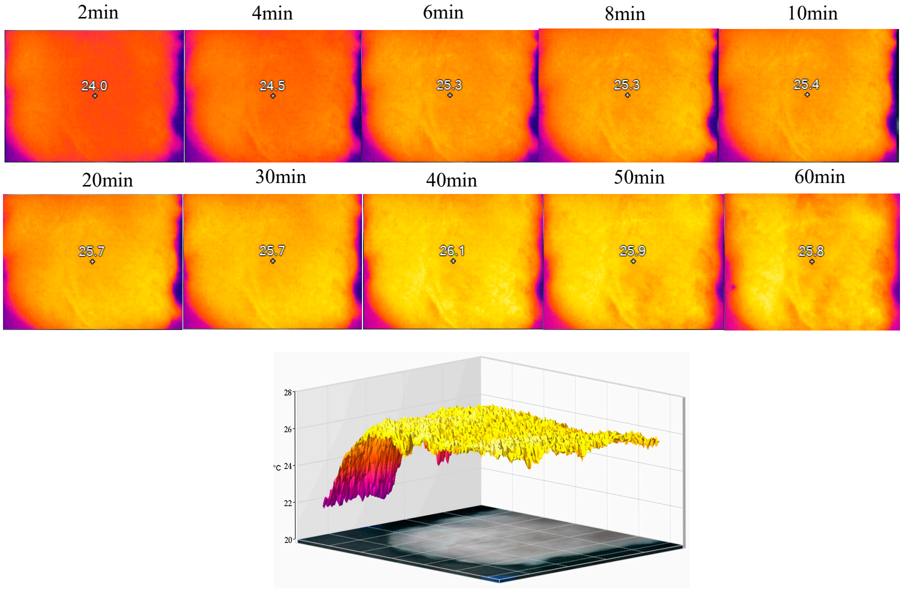

d. Temperature distribution of modified PEA batting during moisture adsorption

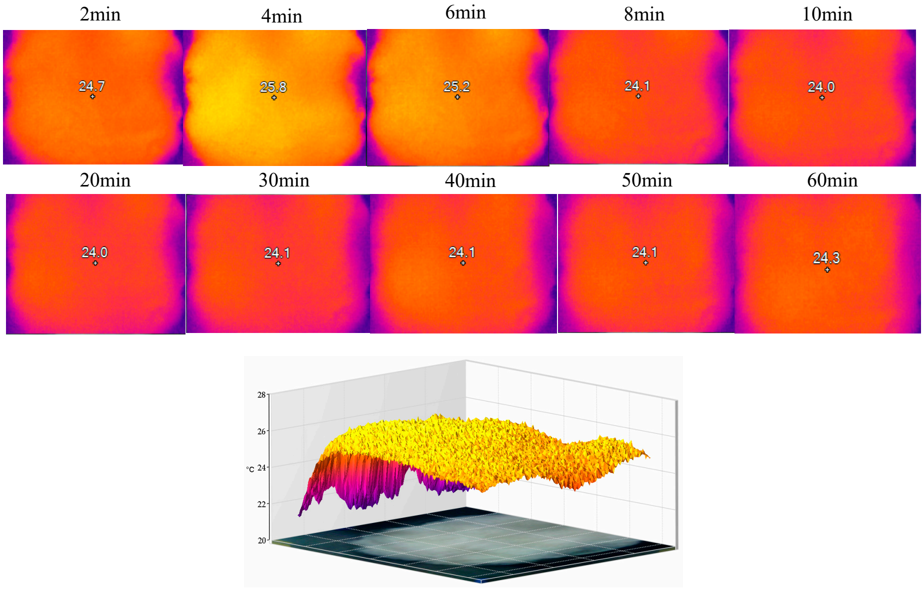

e. Temperature distribution of modified PAN batting during moisture adsorption

Fig.S1. Temperature distribution of different fiber batting during the moisture adsorption

2. Scanning Electronic Image of Fiber Samples

Scanning electron microscope (SEM) image of different samples were illustrated in Fig.S2.

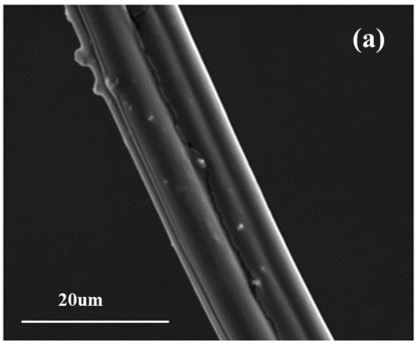

Surface image of collagen protein viscose

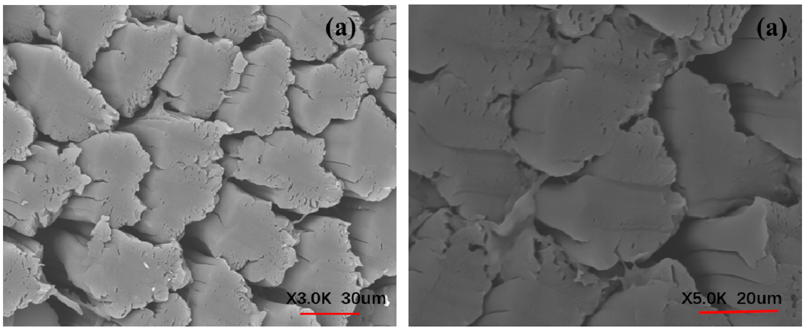

Cross section image of collagen protein viscose

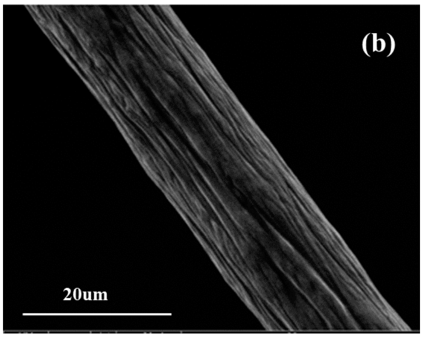

Surface image of modified PAA

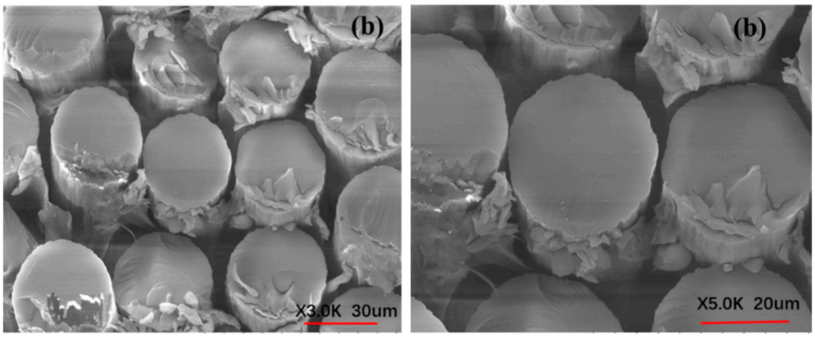

Cross section image of Modified PAA

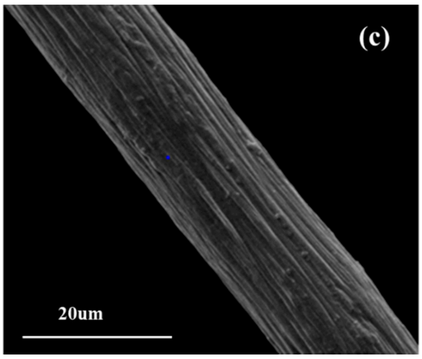

Surface image of modified PEA

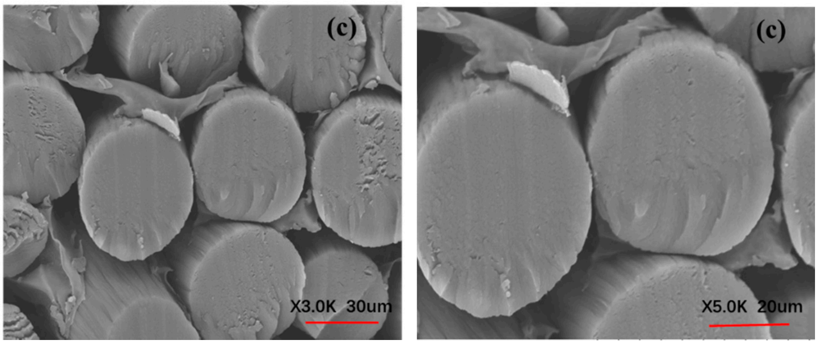

Cross section image of Modified PEA

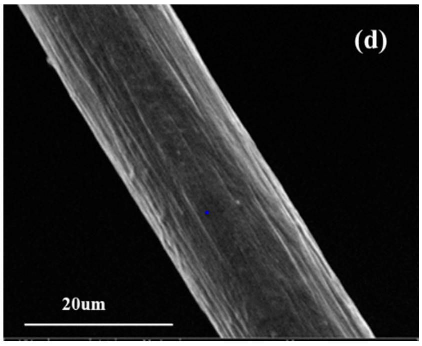

Surface image of modified PAN

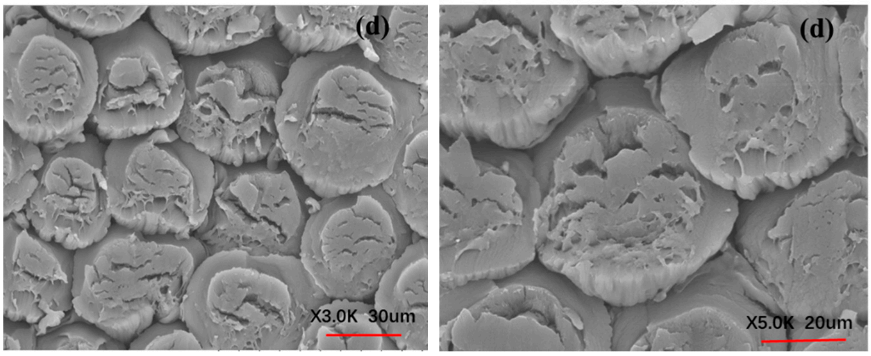

Cross section image of Modified PAN

Fig.S2 surface images and Cross section images of (a)collagen protein viscose, (b)modified PAA, (c)modified PEA, (d)modified PAN.

3. TGA-DSC analysis of absorbed water state on modified fibers

In this study, different forms of adsorbed water presented on high hydrophilic fiber materials were analyzed using TGA-DSC. Three different regions were identified on the TGA-DSC curves. These regions were defined by inflection points from the rate of mass loss and heat flow curves and this helped to get insight on the form of adsorbed water. (the identified regions from TGA-DSC curves of different hygroscopic exothermic fibers showed in Fig. S3 in supplementary material). The region I was assigned to the evaporation of free water from the crucible and water on the surface of the fiber. The region II could be assigned to loosely bound water. The region III was considered to be related to evaporation of tightly bound water.

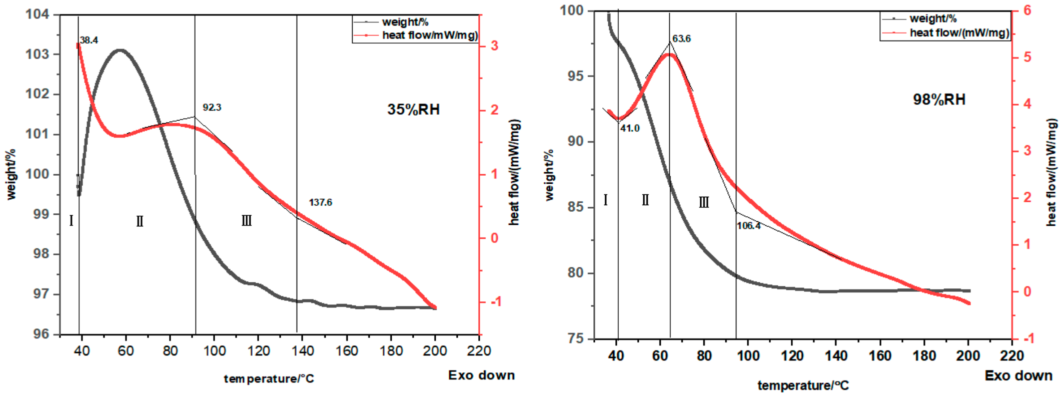

a. TGA-DSC analysis of absorbed water state on collagen protein viscose fiber

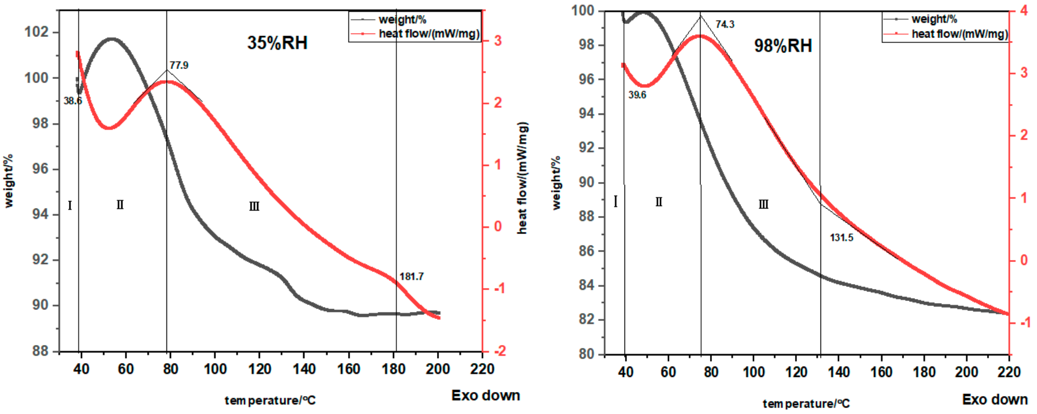

b. TGA-DSC analysis of absorbed water state on modified PAA fiber

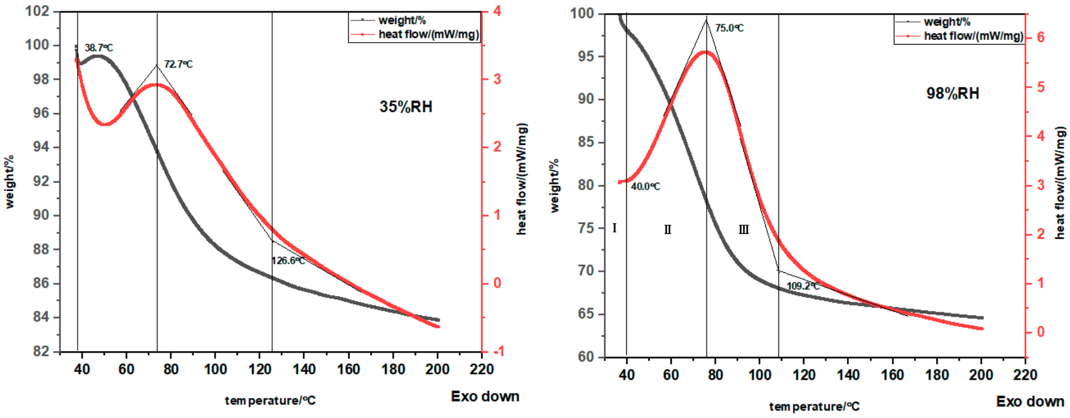

c. TGA-DSC analysis of absorbed water state on modified PEA fiber

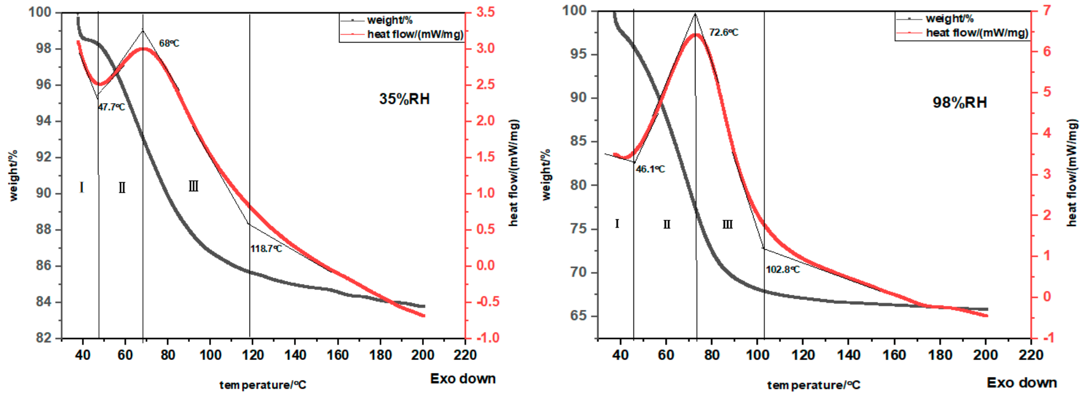

d. TGA-DSC analysis of absorbed water state on modified PAN fiber

Fig.S3. TGA-DSC analysis of absorbed water state on different fibers at 20℃,35% RH and 20℃,98%RH

It could be seen from the Fig.S3. that the weight of these fiber materials exhibited an increasing trend with the increase of temperature at the beginning under the low humidity condition of 35%RH, which was attribute to the low content of free water and moisture adsorption of fiber in the crucible(65%RH). The moisture content of each kind of fiber material in different regions was shown in Table S1.

Table S1 Moisture contents of different water formations on samples at 20°C for 35%RH and 98%RH determined by TGA–DSC analysis.

Material sample	Relative humidity (RH/%)	Free water /%	Loose bond water /%	Tightly bond water /%	Total water content /%
Collagen protein viscose	35	0.48	0.82	1.86	3.16
	98	2.47	10.36	8.01	20.84
modified PAA	35	0.62	1.97	7.74	10.33
	98	0.62	5.63	9.15	15.4
modified PEA	35	1.02	4.34	7.73	13.69
	98	1.93	19.37	10.67	31.97
modified PAN	35	1.81	5.12	7.4	14.33
	98	4.31	18.32	9.42	32.05
